# Assessment of Polyacrylamide Based Co-Polymers Enhanced by Functional Group Modifications with Regards to Salinity and Hardness

**DOI:** 10.3390/polym9120647

**Published:** 2017-11-27

**Authors:** Saeed Akbari, Syed Mohammad Mahmood, Isa M. Tan, Hosein Ghaedi, Onn Lin Ling

**Affiliations:** 1 Centre of Research in Enhanced Oil Recovery (COREOR), Petroleum Engineering Department, Universiti Teknologi PETRONAS, Seri Iskandar, Tronoh 32610, Malaysia; 2 Shale Gas Research Group (SGRG), Institute of Hydrocarbon Recovery, Petroleum Engineering Department, Universiti Teknologi PETRONAS, Seri Iskandar, Tronoh 32610, Malaysia; 3 Fundamental and Applied Science, Universiti Teknologi PETRONAS, Seri Iskandar, Tronoh 32610, Malaysia; isa.mtan57@gmail.com; 4 Chemical Engineering Department, Universiti Teknologi PETRONAS, Seri Iskandar, Tronoh 32610, Malaysia; ghaedi.hosein63@gmail.com; 5 Petroleum Engineering Department, Universiti Teknologi PETRONAS, Seri Iskandar, Tronoh 32610, Malaysia; onnlin.ling@gmail.com

**Keywords:** rheology, AN132 VHM, FLOCOMB, SUPERPUSHER SAV55, THERMOASSOCIATIF, salinity-hardness

## Abstract

This research aims to test four new polymers for their stability under high salinity/high hardness conditions for their possible use in polymer flooding to improve oil recovery from hydrocarbon reservoirs. The four sulfonated based polyacrylamide co-polymers were FLOCOMB C7035; SUPERPUSHER SAV55; THERMOASSOCIATIF; and AN132 VHM which are basically sulfonated polyacrylamide copolymers of AM (acrylamide) with AMPS (2-Acrylamido-2-Methylpropane Sulfonate). AN132 VHM has a molecular weight of 9–11 million Daltons with 32 mol % degree of sulfonation. SUPERPUSHER SAV55 mainly has about 35 mol % sulfonation degree and a molecular weight of 9–11 million Daltons. FLOCOMB C7035, in addition, has undergone post-hydrolysis step to increase polydispersity and molecular weight above 18 million Daltons but it has a sulfonation degree much lower than 32 mol %. THERMOASSOCIATIF has a molecular weight lower than 12 million Daltons and a medium sulfonation degree of around 32 mol %, and also contains LCST (lower critical solution temperature) type block, which is responsible for its thermoassociative characteristics. This paper discusses the rheological behavior of these polymers in aqueous solutions (100–4500 ppm) with NaCl (0.1–10 wt %) measured at 25 °C. The effect of hardness was investigated by preparing a CaCl_2_-NaCl solution of same ionic strength as the 5 wt % of NaCl. In summary, it can be concluded that the rheological behavior of the newly modified co-polymers was in general agreement to the existing polymers, except that THERMOASSOCIATIF polymers showed unique behavior, which could possibly make them a better candidate for enhanced oil recovery (EOR) application in high salinity conditions. The other three polymers, on the other hand, are better candidates for EOR applications in reservoirs containing high divalent ions. These results are expected to be helpful in selecting and screening the polymers for an EOR application.

## 1. Introduction

Water flooding is an improved oil recovery technique that is used to displace oil and maintain reservoir pressure. A common problem in this process is the unstable displacement because the viscosity of injected water is often much lower than the reservoir oil, which causes water to overrun. Addition of polymer to the injected water, called polymer flooding, can successfully alleviate this problem by increasing the viscosity of displacing water, which results in a more favorable oil displacement [1,2,3,4]. This viscosity enhancement makes the front more stable, thus enabling it to push the oil towards production well in a piston-like manner [5].

A suitable polymer for successful polymer flooding in a sandstone reservoir needs to have several specific features. To begin with, it should have high viscosifying power at low dosages to keep the polymer flooding process cost-effective and economical. Polymer structure should also have an adequate thermal stability for the target oil reservoir, e.g., it should not contain –O– in the carbon chain for high-temperature applications. This is because the oxygen in the main backbone can cleave the polymer, therefore, polyoxyethylene, sodium carboxymethyl cellulose should be avoided for high-temperature reservoirs. In addition, its adsorption on the rock surfaces should be low to minimize polymer dilution and economic loss. Polyelectrolyte, i.e., polymers with multiple charges distributed along the chain are a good choice for sandstone reservoirs if the economy is a critical factor. The negatively charged surfaces of sandstone rocks repel polyelectrolytes having negative ionic hydrophilic groups (such as carboxyl groups, –COO^−^), thereby incurring lower adsorption losses. A synergetic effect is also realized when molecules are stretched by chain repulsion that results in an increase in viscosity. The chemical stability is another criterion in screening polymers for a target reservoir. For example, polymers containing the nonionic hydrophilic group, such as amide groups (–CONH_2_), offer better chemical stability in reservoirs containing mono- and divalent ions than pure polyelectrolyte polymers [6].

**Figure 1:**
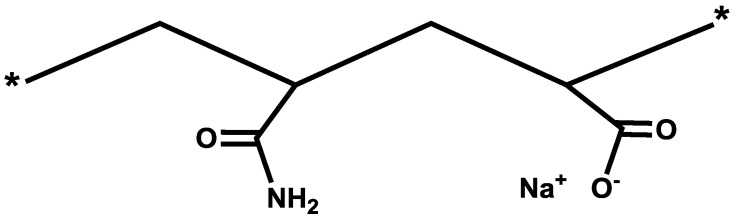
The copolymer of acrylamide and sodium acrylate (HPAM).

In consideration of the above, it appears that partially hydrolyzed polyacrylamides (HPAM), having both amide and carboxyl groups (Figure 1), would be a suitable candidate for polymer flooding applications. The hydrolysis in HPAM, however, significantly increases at temperatures above 70 °C. As the degree of hydrolysis reaches the critical value of 40%, polymer precipitation (due to bridging effect) occurs in the presence of high concentrations of divalent cations [5,7,8,9,10,11]. The degree of hydrolysis is defined here as the fraction of the carboxyl residue replacing acrylamide units over the total number of the polymer macromolecule.

Whereas, the viscosity of polyacrylamide (PAM) as non-ionic polymers is not sensitive to salts/hardness concentrations, the polyelectrolytes like HPAM experience significant changes. The change in viscosity is caused by the interaction between fixed charges along the chain and the mobile ions in solution that leads to the formation of electrical double layers with counterions.

To understand the effect of salinity and hardness on the viscosity of the polymer, it is important to note that the thickness of the electrical double layer is inversely proportional to the ionic strength, *I_s_*, which is a function of the ionic concentration given by [5]:

(1) $$I_{s}=\frac{1}{2}\sum^{\;}m_{i}z_{i}^{2},$$

where *m_i_*; is the molar concentration of the *i*th ion and *z_i_* is its charge.

The flexible polyelectrolytes expand in a solvent of low ionic strength (low salt concentrations) as a result of the mutual repulsion of the charges along the chain and contract in high salt concentrations. In saline solutions, polymer coils or collapses in order to reduce the surface free energy. The size of the polyelectrolyte coil is a function of double layer repulsion and polymer configuration in such a way that the repulsive energy is balanced by the loss of energy due to the change in polymer configuration to a lower energy state (more coiling).

For a flexible polyelectrolyte, such as HPAM, changing the salt concentration leads to dramatic changes in viscosity. Although a typical flexible polyelectrolyte is spherical (on the average), the conformation can change quite easily. HPAM is found to be more favorable for reservoirs with low water salinity, whereas xanthan is more commonly used for higher salinity ones. Since the salinity causes the molecular chain to collapse, viscosity reduction will be more remarkable for HPAM than xanthan. This is explained by the fact that HPAM has a lower degree of rigidity in comparison with xanthan molecules, thus the presence of mono/divalent ions in the solution results in a much smaller molecule of HPAM.

The effect of divalent ions, such as Ca^2+^ and Mg^2+^, is even more significant than that of monovalent species, such as Na^+^ and K^+^. The divalent ions, due to a higher charge and polarizability, bind even more tightly to the polyelectrolytes, therefore, can cause more viscosity reduction as compared to the monovalent ions. Divalent ions can also cause a drop in the viscosity of polymer solution due to the bridging effect.

For oil reservoirs having milder conditions (low temperature, low salinity, and low oil viscosity), many polymers are commercially available for enhanced oil recovery (EOR) applications. However, there is a high demand for new polymers that can sufficiently viscosify the injected water for reservoirs with harsh conditions (high salinity/hardness). Many oilfields in China have high temperatures and salinities, such as “Huabei”, “Zhongyuan”, “Tahe”, and “Tarim”. The reservoir temperatures range from 97.5 to 150 °C, and the salinity of formation water from 84,000 to 284,000 ppm with high concentrations of divalent metal cations [12]. Also, many oil reservoirs in the Middle-East have a high salinity of 200,000 ppm or more [13,14,15]. Malaysian offshore oil fields have high reservoir temperatures of more than 100 °C, and use sea water as injection fluid [16]. In the North Sea, some reservoir brines contain up to 25 percent sodium and calcium chlorides [17]. Under these harsh conditions, polyacrylamides lose their thickening power, and are thus unsuitable. New polymers are needed that can provide adequate thermal and chemical stability with low dosage to economically provide target viscosity in the reservoir. Fortunately, several modified polymer types have emerged that have superior viscosifying power at high salinity/hardness conditions yet resist hydrolysis at high-temperatures. This study investigates four of these modified polymers, which are basically HPAM based copolymers functionalized with 2-Acrylamido-2-Methylpropane Sulfonate (AMPS) monomer.

The co-polymers investigated in this study were FLOCOMB, an anionic post-HPAM [18] with increased molecular weight and polydispersity [19,20], AN132 VHM, a sulfonated polyacrylamide copolymer with a sulfonation degree of 32 mol % and a high molecular weight [19,21,22] SUPERPUSHER SAV55, having a functional groups that are similar to AN132 VHM, but with a higher degree of sulfonation [21] and THERMOASSOCIATIF, a copolymer showing thermo thickening behavior [21,23,24].

Since these co-polymers were developed rather recently (appeared in or after 2015 [20,21,22,23]), their rheological behavior has not been reported in the literature adequately. In this experimental work, their rheological behavior as a function of salinity, hardness, and polymer concentration has been investigated with a special consideration to their viscosifying power in the presence of salinity and hardness. Even though these polymers aim to be applied at high temperatures, this study focused on the investigation at room temperature as a necessary first step towards an understanding of their rheological behavior. The rheological behavior of these polymers at high temperatures was previously reported [4].

## 2. Materials and Methods 

### 2.1. Polymers Descriptions

The Four co-polymers selected for this experiment are briefly described below.

FLOCOMB C7035 is an anionic post-HPAM [18]. Through post-hydrolysis process, molecular weight, and polydispersity (i.e., molecular weight distribution) were increased so as to provide a full range of calcium tolerance thus increasing chemical stability in very hard and salty brines [19,20].

**Figure 2:**
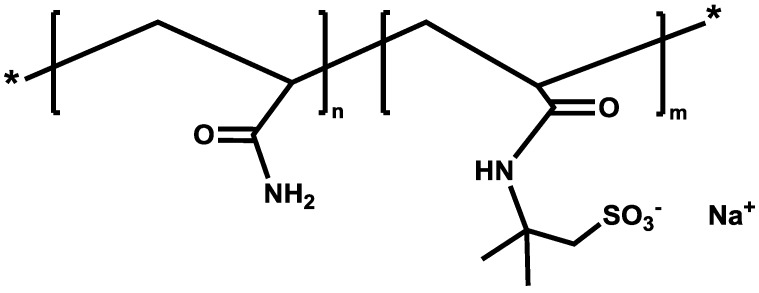
The molecular structure of sulfonated polyacrylamide polymers (AM/AMPS).

AN132 VHM is a sulfonated polyacrylamide copolymer of AM (acrylamide) with AMPS (2-Acrylamido-2-Methylpropane Sulfonate), as shown in Figure 2, with a 32 mol % sulfonation degree, and a high molecular weight [19,21,22]. Polymers with a high degree of sulfonation have shown to be less sensitive to salinity and temperature, thus recommended for applications in the reservoirs of up to 95 °C [19,21,22,23].

SUPERPUSHER SAV55 is similar to AN132 VHM in containing similar functional groups (AM and AMPS), but differs from AN132 VHM mainly in having a higher sulfonation degree, which extends it stability range up to 100 °C [21]. 

THERMOASSOCIATIF is from the family of stimuli-responsive polymers which are capable of associating at specific temperatures. It consists of water-soluble main chains with blocks or side groups with moieties. Several structural modifications were made to the THERMOASSOCIATIF polymer, yet its backbone remains similar to AN132 VHM copolymer. It has shown thermo-thickening behavior, which is often referred to as thermo-responsive [21] or thermosensitive copolymer [21,23]. 

Akbari et al. [4] discussed the properties of this type of polymers in detail. In their study, beyond LCST (lower critical solution temperature), the viscosity increased sharply and peaked at 55 °C for a brine with 5 wt % NaCl and at 65 °C for a CaCl_2_-NaCl mixture. The unique behavior of this polymer can have an economic advantage in a reservoir with harsh conditions because a lower concentration of polymer will be required to attain the viscosity target as compared to other polymers.

**Figure 3:**
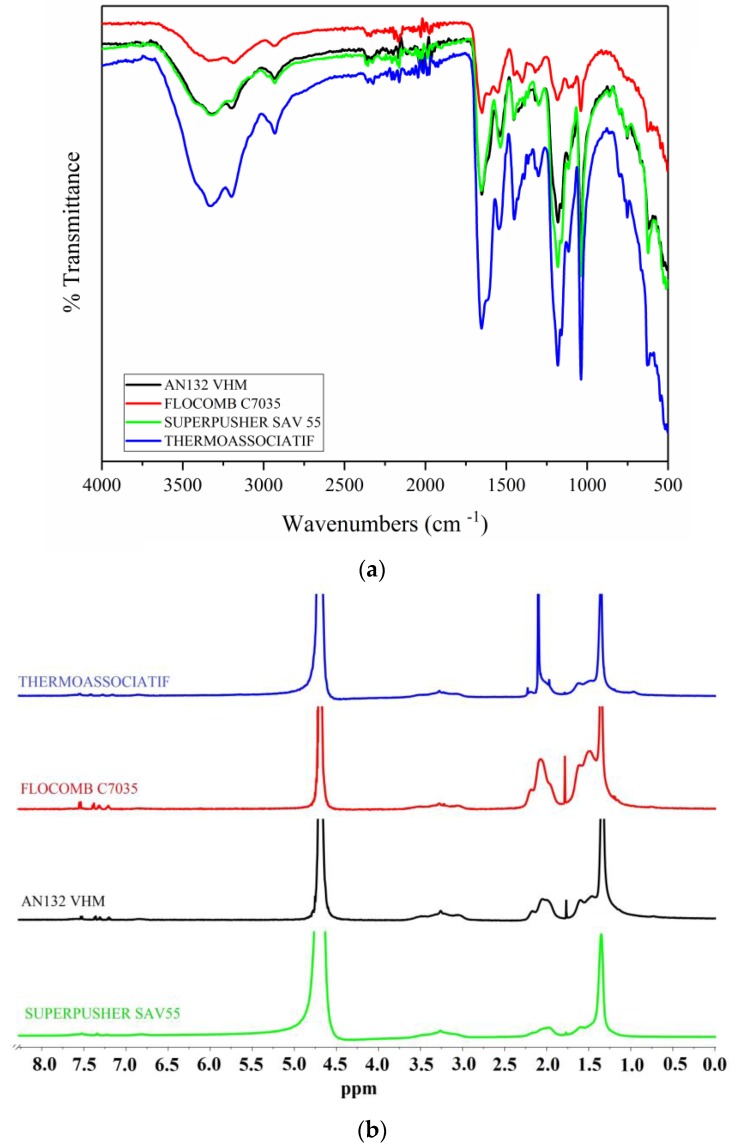
Fourier transform infrared spectroscopy (FTIR) (**a**) and proton nuclear magnetic resonance (^1^H-NMR) (**b**) spectra of copolymers (FTIR and NMR spectra of THERMOASSOCIATIF polymer extracted from our previous work [4]).

Figure 3 presents the FTIR (Fourier transform infrared spectroscopy) and ^1^H-NMR (proton nuclear magnetic resonance) spectra of the copolymers. Nicolet™ iS™ 10 FT-IR Spectrometer (Atkinson, NH, USA) was used for FTIR spectrum using polymer powders, whereas the Bruker 500 NMR spectrometer (Bruker Biospin, Fällanden, Switzerland) was used for conducting NMR of the aqueous solutions of the polymers using D_2_O as a solvent. 

FTIR spectra (Figure 3a) peak at 1652 cm^−1^ may possibly be assigned to the C=O stretching vibrations of the –CONH_2_. Also, wavenumbers 3325 and 2930 cm^−1^ represent the N–H stretching vibrations of the –CONH_2_ and –CH_3_ groups, respectively, whereas 1037 and 1181 cm^−1^ peaks may involve the –SO_3_^−^ group [25]. 

In the NMR spectra (Figure 3b), the peaks between 1.1–1.7 ppm are attributed to the hydrogens in the main chain. The peak at 2.10 ppm may be assigned to the hydrogen atoms of the CH_3_ groups. The peak at about 3.2 ppm may be attributed to the hydrogens of the CH_2_ group bonded to SO_3_Na [26]. The peak at 4.7 ppm is attributed to the hydrogens of the NH and NH_2_ groups, and the water in the solvent D_2_O (99.9%) and in the copolymer [27].

Table 1 provides a list of the tested products along with their characteristics. All four types of the copolymers were thankfully provided in powder form by SNF Floerger (ANDREZIEUX Cedex, Andrézieux-Bouthéon, France).

### 2.2. Polymer Solution Preparation

For samples required for investigating the effect of salinity, a weighed amount of NaCl (sodium chloride) salt was added to deionized water to yield solutions of desired concentrations ranging from 0.1 to 10 wt % (weight percent). For samples required for investigating the effect of divalent ions, a CaCl_2_-NaCl solution (Table 2) was prepared in deionized water with the same ionic strength as of the 5 wt % NaCl solution. The prepared brine sample solutions were stored at 25 °C for later use.

After preparing a brine solution with either NaCl or CaCl_2_-NaCl, it was placed under a propeller stirrer, and rotation at 700 rpm started just before adding polymer powder. This high-speed rotation at the time of polymer addition and the process of slowly adding polymer into the solvent was required to avoid agglomeration of polymer particles, which may be formed if the powder does not become wetted evenly [22,29]. Aluminum foils were used to cover the sample beakers so as to minimize solution contact with air. After 30 min of last polymer addition, the stirrer speed was reduced to medium (300 rpm) and the solution was stirred overnight to assure that the hydration has been completed and the polymer solution has been homogenized. Initially, a polymer solution of 5000 ppm concentration was prepared using the procedure above, and was then diluted to the required concentrations, ranging from 100 to 4500 ppm. 

### 2.3. Viscosity Measurement

For the calculation of critical overlap concentration (C*) that required viscosity measurements at very low polymer concentrations, Anton-Paar rolling-ball viscometer (Lovis 2000 M/ME, Graz, Austria) was used with an appropriate capillary tube. To assure accurate measurements, the viscometer was first calibrated with Millipore water. A suitable capillary tube was then properly cleaned with deionized water and ethanol before and after each run, after which the moisture inside the capillary tube was dried using air blower. After filling the tube with one of the polymers using a 1 mL syringe, a standard ball was inserted into the capillary and viscosity was measured after attaining temperature equilibrium. Each viscosity measurement at atmospheric pressure and 25 °C was repeated thrice and their average value was reported. The measurement uncertainty of the equipment was ±5 × 10^−3^ mPa·s and that of temperature was ±0.02 °C.

For the rest of the measurements, the protocol described by Akbari et al. [4] was adopted. Polymer solutions, as soon as they were prepared, were brought for viscosity measurement using a rheometer. Rheological profiles were obtained using a Bohlin Gemini 2 (Malvern Instruments, Malvern, UK) with cone-plate geometry (1°, 4 cm). 

## 3. Results and Discussion

### 3.1. Shear Rate Dependency of Polymer Viscosity

**Figure 4:**
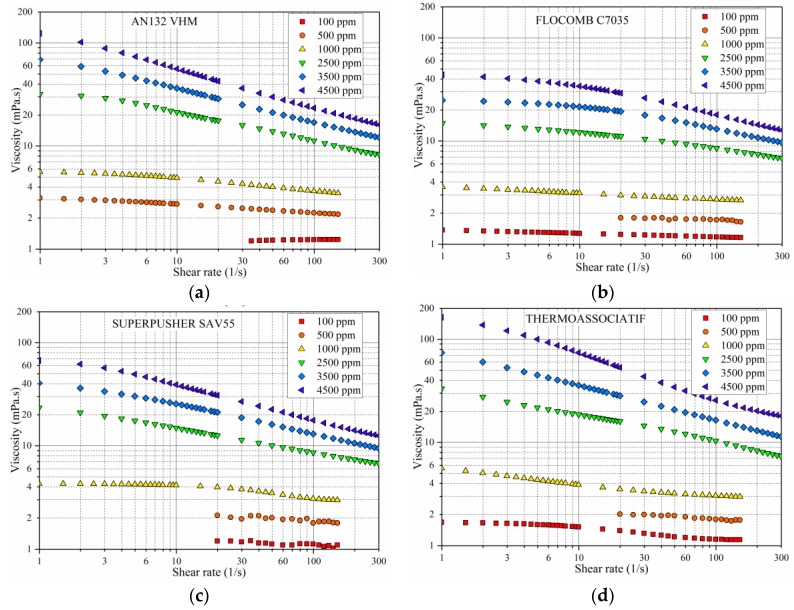
The effect of polymer concentration on the rheological behavior of polymer solutions prepared in 5 wt % NaCl and measured at 25 °C for four copolymers (**a**) AN132 VHM, (**b**) FLOCOMB C7035, (**c**) SUPERPUSHER SAV55, and (**d**) THERMOASSOCIATIF.

For variable polymer concentrations (100–4500ppm), prepared in 5 wt % NaCl, and measured at 25 °C, the following observations were made from Figure 4:

At concentrations below 1000 ppm, all four of the polymers behave somewhat as Newtonian fluids, i.e., the viscosity was almost independent of shear rate. This behavior was observed in “dilute” polymer solutions only because in this condition the chains are separated from each other behaving more or less independently, while primarily interacting only with the solvent molecules [30].

At concentrations above 1000 ppm, all four polymers behave as pseudoplastic (shear thinning). This could be explained by the alignment of the polymer molecules under the application of the shear. This molecular alignment will allow for easier flow of the molecules reducing the viscosity at higher shear rates [22,31].

The pseudoplasticity (shear-rate dependency) of all four polymers increased with an increase in polymer concentration. This phenomenon is attributed to an increase of macromolecular chain entanglement, causing higher viscosity, and thereby extending shear thinning region [22,32]. 

Viscosity profile for a higher value of shear rates shows that viscosity is approaching upper Newtonian regime possibly because the polymer coils and their distortion is approaching the maximum.

As per expectations, the overall rheological behavior seen in this part of the study was similar to the ones previously observed by other researchers who have studied bulk rheology of polyelectrolyte solutions [33].

**Figure 5:**
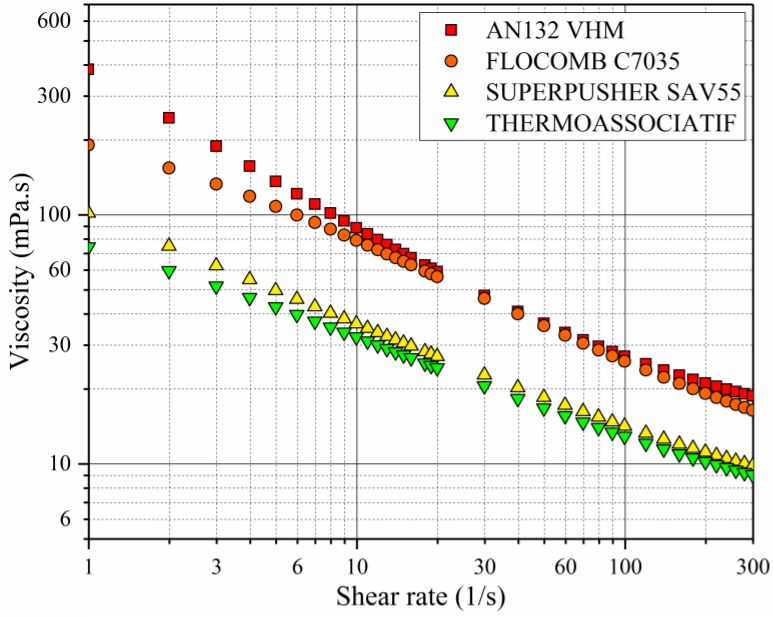
The effect of polymer type on the rheological behavior of solutions prepared in 0.1 wt % NaCl, 2500 ppm polymer concentration, and measured at 25 °C.

For polymer solutions of 2500 ppm polymer concentration, prepared in 0.1 wt % NaCl, and measured at 25 °C, the following observations were made from Figure 5:

At low shear rates, the viscosity of the four polymers was in the following decreasing order: AN132 VHM (highest), FLOCOMB C7035, SUPERPUSHER SAV55, THERMOASSOCIATIF (lowest). A comparison of viscosity profiles of AN132 VHM with SUPERPUSHER SAV55, and FLOCOMB C7035 with THERMOASSOCIATIF reveals that an increase in molecular weight enhanced polymer viscosity provided the anionicity that was about the same. This can be explained by the fact that higher molecular weight corresponds to a higher chain size, which facilitates chain interaction and causes a higher viscosity [22,34].

At higher shear rates above 20–30 s^−1^, the difference between the four polymers became insignificant. Viscosity difference that is observed in low shear rate is mainly because of random coils moving past each other without coil deformation depending upon polymer characteristics. But at higher shear rates, the coils are deformed and slip past each other more easily and role of polymer characteristics and their interaction becomes minimized. Therefore, shear rate value becomes the dominant factor in the determination of viscosity value.

**Figure 6:**
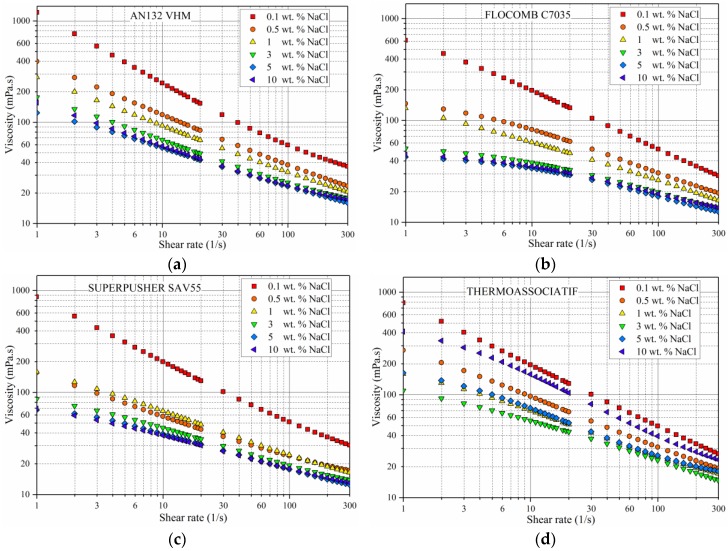
The effect of salinity on the rheological behavior of polymers solution prepared with 4500 ppm polymer concentration and measured at 25 °C for four copolymers (**a**) AN132 VHM, (**b**) FLOCOMB C7035, (**c**) SUPERPUSHER SAV55, and (**d**) THERMOASSOCIATIF.

For polymer solutions of 4500 ppm polymer concentration, prepared in varying salinities and measured at 25 °C, the following observations were made from Figure 6.

The viscosity and pseudoplasticity of three of the four polymers decreased with an increase in salinity. Both viscosity and pseudoplasticity reductions were very pronounced at low salinities (0.1 wt % NaCl), but as the solution salinity increased, these changes became subdued. This behavior has been reported earlier [22], and is supported by the fact that by increasing salinity, most of the negative charges on polymer chain will be neutralized by Na^+^ ions shrinking the polymer macro-molecule (coiling) and ultimately lowering viscosity [5,6]. 

THERMOASSOCIATIF polymer showed similar behavior up to 3 wt % NaCl concentration, i.e., the viscosity and pseudoplasticity decreased with an increase in salinity. However, this behavior was reversed as salinity increased above 3 wt % NaCl concentration, such that the viscosity and pseudoplasticity increased with an increase in salinity. This behavior will be discussed later when considering more evidence (from Figure 10).

### 3.2. The Effect of Polymer Concentration on the Polymer Viscosity

Low polymer concentration solutions act as an ideal solution because polymer molecules or spheres are separated from each other. When polymer concentration is increased, these spheres or molecules become congested and ultimately begin to touch each other. This specific concentration is termed as overlap concentration (C*). At C*, spheres are distributed all over the solution and pack the whole volume. Thus, at *C* > C*, a semi-dilute regime exists, in which chains are overlapping and entangled. Consequently, their mobility is greatly reduced as compared with the chains in dilute solutions where *C* < C* [5,6]. A quantitative definition of C* can be given by using the intrinsic viscosity [η] concept and is presented in Equation (1) [30,35]:

(2) $$C^{*}\left[\eta \right]≅1$$

(3) $$\left[\eta \right]=\underset{c\to 0}{lim}\frac{\eta -\eta_{s}}{c\eta_{s}}$$

where *c* is the concentration of polymer (ppm) $\eta_{s}$ is the viscosity of pure solvent (mPa·s), and η is the viscosity of the polymer solution (mPa·s). 

**Figure 7:**
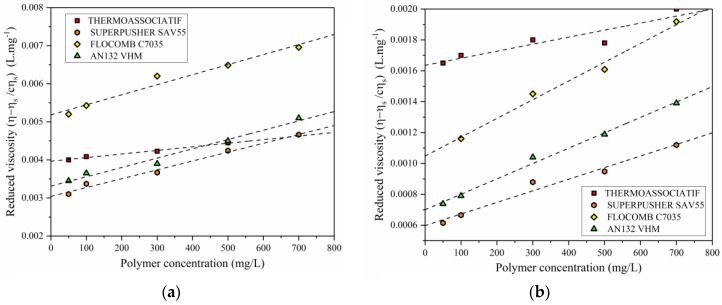
Determination of intrinsic viscosity of copolymer samples using data plotted as reduced viscosity versus concentration for a brine with (**a**) 0.1 wt % salinity and (**b**) 5 wt % salinity.

**Figure 8:**
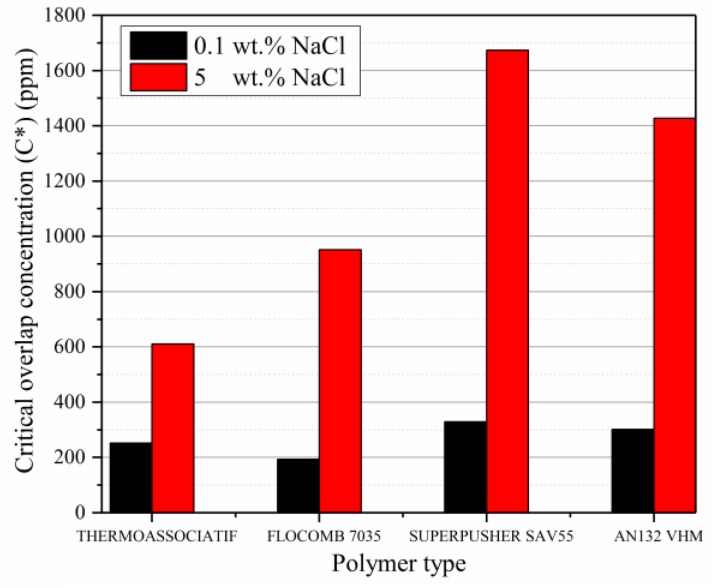
Critical overlap concentration (ppm) as a function of salinity for the studied polymers at 25 °C.

To see the effect of salinity on critical overlap concentration (C*), solutions of low polymer concentration were prepared with fixed NaCl concentrations of 0.1 and 5 wt %, and their viscosity was measured at 25 °C. The intrinsic viscosity was computed using the Equation (3) with the help of the data plotted in Figure 7 and was extrapolated to zero. The C* was then computed using Equation (2) and results are plotted in Figure 8, and show the following: 

By increasing the salinity from 0.1 to 5 wt % of NaCl, the C* was increased for all polymer types. It is because, by increasing salinity, more Na^+^ will be available to neutralize the negative charges on polymer chain, resulting in a lower repulsion among them. To attain the level of repulsion corresponding to C*, a higher concentration of polymers will be required [22].

When the data in Figure 8 is viewed in conjunction with Table 1, increasing both molecular weight and anionicity will lower C*. This is consistent with previous findings in the literature [22]. Increasing molecular weight will result in lower C* because it directly affects the polymer chain size, and consequently the radius of gyration of the polymer. In terms of anionicity, this reduction can be explained by the fact that extension of the polymer molecule in the solution is a function of charge density of the polymer. More negative charges result in more extensions, which can facilitate an interaction between the polymer chains in lesser polymer concentration.

**Figure 9:**
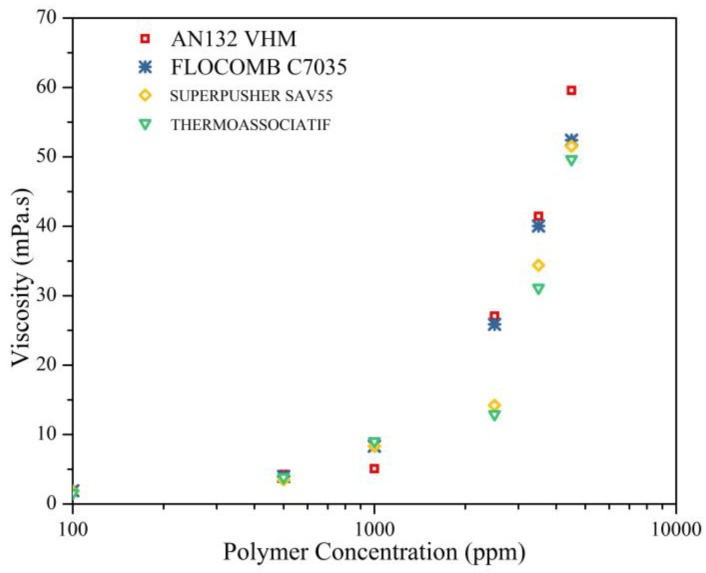
The effect of polymer concentration on the viscosity of solution prepared in 0.1 wt % NaCl measured at 25 °C and at 100 s^−1^ shear rate.

To see the effect of polymer concentration on the viscosity of polymers, solutions with variable polymer concentrations (100–4500 ppm) were prepared in 0.1 wt % NaCl, and were measured at 25 °C and at 100 s^−1^ shear rate. The following observations can be made from Figure 9, which presents the measurement data:

The viscosity is a strong function of polymer concentration [36] in general. For each of the four type of polymers, a higher viscosity was observed at higher polymer concentrations. This is because more chains are available at higher concentrations, thus increasing the probability of interaction and entanglement between polymer chains [37].

There appears to be a specific polymer concentration beyond which the rate of change in viscosity suddenly increases. At concentrations below 1000 ppm, the increase in viscosity with increasing concentration is rather slow, but after 2000 ppm, a slight change in concentration increases viscosity rather significantly. At this specific concentration, polymer regimes change from “semi-dilute un-entangled” to “semi-dilute entangled”. The later regime involves a more significant increase in viscosity [35].

### 3.3. Effect of NaCl Concentration on the Polymer Viscosity

**Figure 10:**
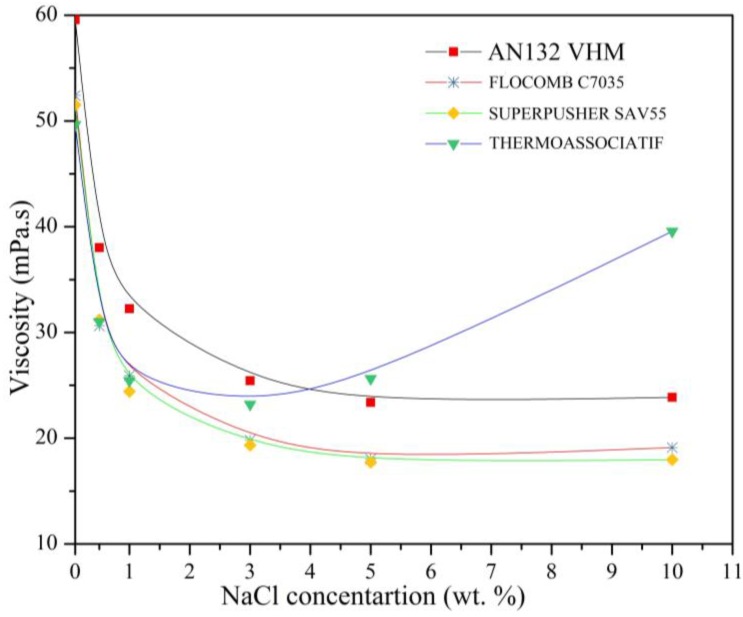
The effect of salinity on the viscosity of solution prepared with 4500 ppm polymer concentration, measured at 25 °C and at 100 s^−1^ shear rate.

To get a better understanding of the effect of salinity on the viscosity of polymers at relatively high shear rates of 100 s^−1^, solutions were prepared with 4500 ppm polymer concentration in varying salinities, and their viscosity was measured at 25 °C. The following observations can be made from Figure 10, which represents the measurement data:

The viscosity of three of the four polymers (all except THERMOASSOCIATIF) decreased with an increase in salinity. Viscosity reduction was very pronounced at low salinities (0.1 wt % NaCl), but as the solution salinity increased, these changes became subdued. As discussed previously, the polymer coil shrinks by increasing NaCl concentration in the polymer solution [38]. Therefore, the addition of NaCl reduces the viscosity of polyacrylamide solution significantly. It must be stated that after a certain level of salinity, the further decrease in viscosity slows down and ultimately levels off. The main reason for this behavior is that almost all of the charges on the polymer chain have been neutralized by counter ions of Na^+^.

The viscosity of three of the four polymers (all except for THERMOASSOCIATIF) decreased with an increase in salinity. Viscosity reduction was very pronounced at low salinities (0.1 wt % NaCl), but as the solution salinity increased, these changes became subdued. As discussed previously, the polymer coil shrinks by increasing NaCl concentration in the polymer solution [38,39,40]. Therefore, the addition of NaCl reduces the viscosity of the polyacrylamide solution significantly. It must be stated that after a certain level of salinity, the further decrease in viscosity slows down and ultimately levels off. The main reason for this behavior is that almost all of the charges on polymer chain have been neutralized by counter ions of Na^+^. 

**Figure 11:**
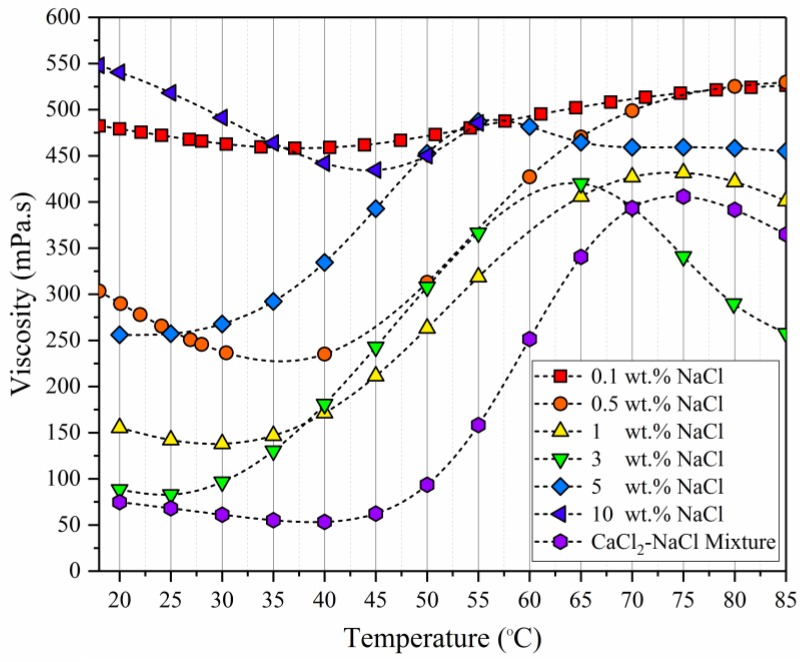
Comparison of rheological profiles of the THERMOASSOCIATIF copolymer at 4500 ppm polymer concentration as a function of temperature and brine salinity at a shear rate (3 s^−1^) (Bohlin rheometer, cone and plate, 2°, 55 mm).

The THERMOASSOCIATIF polymer’s sensitivity to salinity showed similar behavior up to 3 wt % NaCl concentration, i.e., the viscosity decreased with an increase in salinity. However, this behavior was reversed as salinity increased above 3 wt % NaCl concentration such that the viscosity began to increase with an increase in salinity. It has been referred to structural modifications that give its thermosensitivity [23]. This can be explained by the fact that at low salinity concentration, negative charges of the sulfonated group are dominating factor and can cause a great repulsion between polymer chains. By increasing the salinity, more sulfonated groups will be shielded and their effect will be minimized. One of the characteristics of THERMOASSOCIATIF polymers containing LCST-type blocks is that the LCST decreases to a lower temperature with an increase in salt concentration. This is clearly seen in Figure 11 from 0.1 to 3 wt % NaCl concentration. This is due to the fact that addition of salts will partially disrupt the polymer-water interaction and decrease the LCST [41].

By increasing salinity beyond the point where all of the sulfonated groups have been shielded, blocks that were induced during structural modifications have a chance to interact with other blocks of other polymer chains and hence increase solution viscosity. A similar explanation was reported for amphoteric hydrophobically associating polyacrylamide [42]. This kind of behavior in highly saline water makes the THERMOASSOCIATIF polymer more economical because a lower polymer consumption will be required to attain target viscosity for high salinity reservoir applications.

### 3.4. Effect off Divalent Ions Concentrations on the Polymer Viscosity

**Figure 12:**
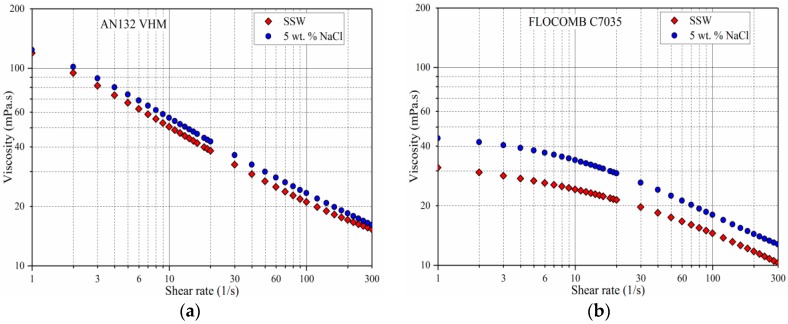

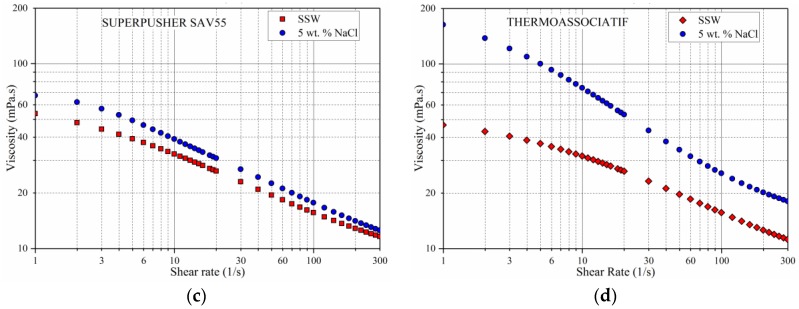
Rheological behavior for 4500 ppm co-polymers in two solvents of same ionic Strength (5 wt % NaCl and CaCl_2_-NaCl) for four copolymers (**a**) AN132 VHM, (**b**) FLOCOMB C7035, (**c**) SUPERPUSHER SAV55, and (**d**) THERMOASSOCIATIF.

To see the effect of divalent ions on the rheology of polymer solutions of 4500 ppm polymer concentration, the samples were prepared in two solvents with the same ionic strength (5 wt % NaCl and CaCl_2_-NaCl) and measured at 25 °C. The following observations were made from Figure 12:

The presence of divalent ions reduced the viscosity as compared with the solution containing only monovalent ions for all of the polymers. It is because cations have more charge screening ability than monovalent, and besides they can also cause ionic bridges [22,23,43].

The viscosity reduction that is caused by divalent ions was very significant in THERMOASSOCIATIF polymer, whereas the other three polymers showed only a small reduction. It is possibly because the THERMOASSOCIATIF polymers coiled up more in comparison to other polymers due to the ion bridging effect caused by divalent ions. It can also be concluded that polymer flexibility of THERMOASSOCIATIF is higher than the other polymers in this study.

The difference of viscosity between mono and divalent ionic solutions was more pronounced at low shear rates. At higher shear rates of 300 s^−1^, however, the difference was negligible. It may be because—at high shear rates—the effect of the alignment and distortion of highly anisotropic chains on viscosity is a lot more dominant than the effect of polymer coiling due to the ion types [31].

## 4. Conclusions

The effect of polymer concentration, salinity, and hardness on the rheology of four newly developed copolymers was studied at 25 °C. These polymers were AN132VHM (medium *M*_W_ [molecular weight], high anionicity, high sulfonation degree), FLOCOMB C7035 (high *M*_W_, medium anionicity, low sulfonation degree), SUPERPUSHER SAV55 (low *M*_W_, high anionicity, sulfonation degree), and THERMOASSOCIATIF (medium M_W_, medium anionicity, medium sulfonation degree). The conclusions of this study are as follows:

- All polymers experienced viscosity loss due to the addition of both mono- and divalent ions, although the THERMOASSOCIATIF polymer showed a uniquely complex salt-responsive behavior in salt concentrations above 3 wt %, whereas it showed thermoassociative characteristics at all of the salinities.
- Out of the four polymers tested, THERMOASSOCIATIF was the most resistant to monovalent ions hence a likely candidate for polymer flooding of high salinity reservoirs.
- Replacing a monovalent ion with a divalent ion while keeping the ionic strength constant did not influence the rheological behavior of THERMOASSOCIATIF polymer, except for significantly lowering its viscosity, thus caution must be exercised when considering it for EOR applications to reservoirs containing brines with divalent ions. Other three polymers showed a higher chemical stability in the presence of divalent ions as their viscosity reduction was small.
- All of the polymers showed shear thinning behavior (pseudoplasticity) at high polymer concentrations. The lower the polymer concentration, the lower was the pseudoplasticity that was ultimately approaching the Newtonian behavior at very low concentrations.
- All of the polymers showed an increase in C* (critical overlap concentration), with an increase in salinity from 0.1 to 5 wt % of NaCl. By the same token, the C* lowered with an increase in molecular weight and/or anionicity.

## Figures and Tables

**Table 1:** Polymer characteristics (extracted from our previous work [4]).

Polymer product (trade name)	Molecular weight (million daltons)	Sulfonation degree (mol %)	Anionicity
FLOCOMB C7035	Very High(>18)	low	Medium
AN132 VHM	Medium (9–11 ^1^)	Medium (32)	High
SUPERPUSHER SAV55	Low (5–7 ^1^)	high	High
THERMOASSOCIATIF	Medium (<12)	medium	Medium

^1^ Data has been provided by SNF Floerger, except the numerical value (indicated by 1) which was extracted from SPE-177073-MS [28].

**Table 2:** CaCl_2_-NaCl Solution Composition.

Solvent	Amount in g/kg of solvent
NaCl ^1^	20
CaCl_2_^1^	19

^1^ Note: Sodium chloride and calcium chloride were provided by J.T. Baker (Phillipsburg, NJ, USA).
